# WS_(1−*x*)_Se*_x_* Nanoparticles Decorated Three-Dimensional Graphene on Nickel Foam: A Robust and Highly Efficient Electrocatalyst for the Hydrogen Evolution Reaction

**DOI:** 10.3390/nano8110929

**Published:** 2018-11-08

**Authors:** Sajjad Hussain, Kamran Akbar, Dhanasekaran Vikraman, Rana Arslan Afzal, Wooseok Song, Ki-Seok An, Ayesha Farooq, Jun-Young Park, Seung-Hyun Chun, Jongwan Jung

**Affiliations:** 1 Graphene Research Institute, Sejong University, Seoul 05006, Korea; hussain@sejong.ac.kr; 2 Institute of Nano and Advanced Materials Engineering, Sejong University, Seoul 05006, Korea; raafzal@hotmail.com (R.A.A.); jyoung@sejong.ac.kr (J.-Y.P.); 3 Department of Physics, Sejong University, Seoul 05006, Korea; mohkamranakbar@gmail.com (K.A.); schun@sejong.ac.kr (S.-H.C.); 4 Department of Energy Science, Sungkyunkwan University, Suwon 16419, Korea; 5 Division of Electronics and Electrical Engineering, Dongguk University-Seoul, Seoul 04620, Korea; v.j.dhanasekaran@gmail.com; 6 Thin Film Materials Research Center, Korea Research Institute of Chemical Technology, Daejeon 34114, Korea; wssong@krict.re.kr (W.S.); ksan@krict.re.kr (K.-S.A.); 7 Department of Physics, COMSATS IIT, Islamabad 45550, Pakistan; aysha_farooq@comsats.edu.pk

**Keywords:** hydrogen evolution reaction, electrocatalysts, electrocatalytic activity, TMDC

## Abstract

To find an effective alternative to scarce, high-cost noble platinum (Pt) electrocatalyst for hydrogen evolution reaction (HER), researchers are pursuing inexpensive and highly efficient materials as an electrocatalyst for large scale practical application. Layered transition metal dichalcogenides (TMDCs) are promising candidates for durable HER catalysts due to their cost-effective, highly active edges and Earth-abundant elements to replace Pt electrocatalysts. Herein, we design an active, stable earth-abundant TMDCs based catalyst, WS_(1−*x*)_Se*_x_* nanoparticles-decorated onto a 3D porous graphene/Ni foam. The WS_(1−*x*)_Se*_x_*/graphene/NF catalyst exhibits fast hydrogen evolution kinetics with a moderate overpotential of ~−93 mV to drive a current density of 10 mA cm^−2^, a small Tafel slope of ~51 mV dec^−1^, and a long cycling lifespan more than 20 h in 0.5 M sulfuric acid, which is much better than WS_2_/NF and WS_2_/graphene/NF catalysts. Our outcomes enabled a way to utilize the TMDCs decorated graphene and precious-metal-free electrocatalyst as mechanically robust and electrically conductive catalyst materials.

## 1. Introduction

Water splitting is widely considered to be an effective route for renewable, clean, and efficient energy production from the abundant water on Earth. Electrocatalytic or photocatalytic water splitting into oxygen and hydrogen may potentially address the global environmental pollution and energy crisis [1,2]. Platinum (Pt) has proved to be a most efficient hydrogen evolution reaction (HER) catalyst, however, it has low appeal to use in industrial applications due to its high cost and scarcity [3]. The development of an inexpensive, Earth-abundant, highly active, and acid-stable material to use as an electrocatalyst is a grand challenge. In recent years, tremendous effort has been made to develop efficient HER catalysts from Earth-abundant materials with lots of active edges to replace Pt, such as transition-metal-based oxides/hydroxides, non-oxides, including metal based sulfides [4,5,6], selenides [7,8,9], carbides [10,11], phosphides [12,13], borate [14], phosphate [15], and their alloys. However, so far most of the catalysts exhibit inferior efficiency compared to Pt, while many processes involve complicated material synthesis and multiple steps, which may result in the increase of cost and further limit potential applications. Graphene is a well-known material, and it has potential for use in various electrocatalyst applications, which include supercapacitor, HER, and DSSCs [16,17]. Recently, tungsten disulfide (WS_2_), which is from the family of transition metal dichalcogenides (TMDCs), has been studied elaborately as an electrocatalyst due to its high electrocatalytic properties [18]. Various studies have been done to promote the electrocatalytic activity of WS_2_ with the combination of highly conductive materials, such as macro- and meso-porous carbon materials, gold (Au), and carbon paper in a hybrid nature for oxygen and hydrogen evolution reactions (OER and HER). Davodi and co-workers [19,20] reported the nitrogen doped multi-walled carbon nanotube (MWCNT) and Ni@γ-Fe_2_O_3_/MWCNTs functionalized with nitrogen-rich emeraldine salt for alkaline HER and OER processes. Luo et al. also demonstrated Fe_3_O_4_@NiFe*_x_*O*_y_* core−shell nano-heterostructures toward the OER with an overpotential −410 mV@1 mA cm^−2^ and Tafel slope of 48 mV dec^−1^ [21]. Zhou et al. [22] used 3D hybrids of WS_2_/graphene/Ni foam as a catalyst in HER application and observed the low overpotential of −119 mV@10 mA cm^−2^, the small Tafel slope of ~43 mV dec^−1^, and the large cathodic current density. Recently, Zhou et al. has reported ternary tungsten sulfoselenide (WS_2(1−*x*)_Se_2*x*_) particles with a 3D porous metallic NiSe_2_ foam to have excellent catalytic performance with −88 mV@10 mA cm^−2^ of overpotential, 46.7 mV dec^−1^ of Tafel slope, and 214.7 μA cm^−2^ of exchange current density [23]. Our group has recently demonstrated a facile way to prepare a MoS_2_ QDs film using a solution process and WS_2_/CoSe_2_ heterostructure for HER applications [24,25]. Moreover, ternary alloys of MoS_2(1−*x*)_ Se_2x_ and WS_2(1−*x*)_ Se_2*x*_ were synthesized as a electrocatalyst for HER by a sputtering-CVD process with the overpotentials of −141 and −167 mV to drive 10 mA cm^−2^ and Tafel slopes of 67 and 107 mV dec^−1^, respectively [26].

Recently, many efficient strategies to increase the number of active edge sites with large surface areas, high porosity, and better intrinsic electrical conductivity or the contact between the catalyst and the electrode were adopted to increase the electrocatalytic activity of electrode material. Herein, we utilized a 3D porous structure nickel (Ni) foam (NF) as a highly conductive skeleton, and produced WS_2_/NF, WS_2_-decorated graphene/NF, and WS_(1−*x*)_Se*_x_* nanoparticles-decorated graphene/NF catalysts for HER applications. The obtained electrodes exhibited low overpotentials of −145, −115, and −93 mV vs. RHE for WS_2_/NF, WS_2_/Graphene/NF, and WS_(1−*x*)_Se*_x_*/graphene/NF, respectively, at 10 mA cm^−2^. The small Tafel slope was obtained for WS_(1−*x*)_Se*_x_*/graphene/NF (51 mV dec^−1^) as compared to the WS_2_/NF and WS_2_/graphene/NF (62 and 63 mV dec^−1^, respectively).

## 2. Experimental Details

### 2.1. Synthesis of Graphene/NF, WS_2_/Graphene/NF, and WS_(1−x)_Se_x_/Graphene/NF

Initially, the Ni foam (NF) was cleaned with ultrasonic baths of acetone, ethanol, and deionized water, baked at 120 °C for 5 min, and then annealed with a hydrogen (H_2_)/argon (Ar) (30/50 sccm) environment at 900 °C for 30 min in a quartz tube furnace to clean the surface of the Ni foam without breaking the vacuum. Then to prepare the graphene on NF, the mixture of H_2_/Ar/methane (CH_4_) flow (H_2_/Ar/CH_4_ = 50:100:50 sccm) was maintained for 30 min. Subsequently, the H_2_/CH_4_ flow gas channel was shut off, and then rapidly cooled to room temperature in Ar environment. Furthermore, prepared graphene/NF was used as substrate for the growth of WS_2_ and WS_(1−*x*)_Se*_x_*.

For WS_2_ growth, ammonium tetrathiotungstate ((NH_4_)_2_WS_4_) (Sigma Aldrich, 99.97%) was used as the main source material. First, a precursor of (NH_4_)_2_WS_4_ (0.2 g) was dissolved in *N*,*N*-Dimethylformamide (DMF) (20 mL), and then the solution was sonicated for 30 min. The 3D graphene/Ni foam was immersed into the prepared (NH_4_)_2_WS_4_ solution and then baked at 100 °C for 30 min. Finally, synthesized films were placed in an annealing chamber and heated up to 450 °C for 30 and 45 min in a sulfur or sulfur/selenium environment to form WS_2_/Graphene/NF and WS_(1−*x*)_Se*_x_*/Graphene/NF. The gas flow Ar/H_2_ flux (50/50 sccm) was maintained, and the pressure of chamber was kept at 2 × 10^−2^ Torr. The same quantity of sulfur/selenium (0.3/0.3 g) powder was used.

### 2.2. Electrochemical Measurements

The electrochemical measurements were conducted in a three-electrode setup with a Biologic SP-300 workstation. The polarization curves were collected using a linear sweep voltammetry (LSV) with a scan rate of 10 mV.s^−1^ in 0.5 M H_2_SO_4_ electrolyte at room temperature. For the LSV measurement, a saturated calomel reference electrode (SCE) was used as the reference electrode. WS_2_(30 min)/NF, WS_2_(45 min)/NF, WS_2_/graphene/NF, and WS_(1−*x*)_Se*_x_*/graphene/NF were used as the working electrode. Also, a graphite rod was used as the counter electrode. All LSV measurements were probed in terms of SCE and then converted to an reversible hydrogen electrode (RHE) scale with the help of the following equation: E(RHE) = E(SCE) + E°(SCE) + 0.059 pH. Electrochemical impedance spectroscopy (EIS) measurements were carried out in a potentiostatic mode with a frequency range from 0.01 Hz to 100 kHz under an amplitude of 10 mV. All the LSV polarizations were recorded after the ohmic drop iR correction. The stability measurement was examined using a chronoamperometric analysis.

## 3. Results and Discussion

**Figure 1:**
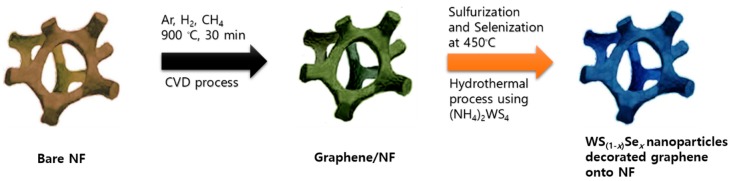
Schematic preparation process of graphene and the WS_(1−*x*)_Se*_x_*/graphene/NF catalyst.

Initially, the graphene was grown on a 3D NF using a chemical vapor deposition (CVD), as reported previously [27]. WS_2_ was further grown by a hydrothermal process using an (NH_4_)_2_WS_4_ precursor on 3D graphene with NF. To improve the crystalline quality, the film was further annealed at 500 °C in a sulfur environment at 30~45 min to form WS_2_ nanoparticle-decorated graphene on NF (WS_2_/graphene/NF). To form WS_(1−*x*)_Se*_x_* nanoparticles-decorated graphene on NF (WS_(1−*x*)_Se*_x_*/graphene/NF), a CVD deposited film was annealed at 450 °C in a sulfur and selenium environment at 30 min, respectively, to form WS_(1−x)_Se_x_ onto graphene. The schematic representation is given in Figure 1.

**Figure 2:**
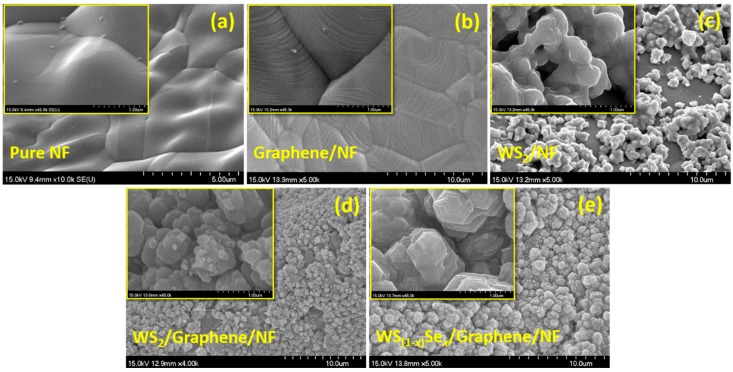
SEM micrographs of (**a**) pure NF; (**b**) graphene/NF; (**c**) WS_2_(45 min)/NF; (**d**) WS_2_/graphene/NF and (**e**) WS_(1−*x*)_Se*_x_*/graphene/NF.

**Figure 3:**
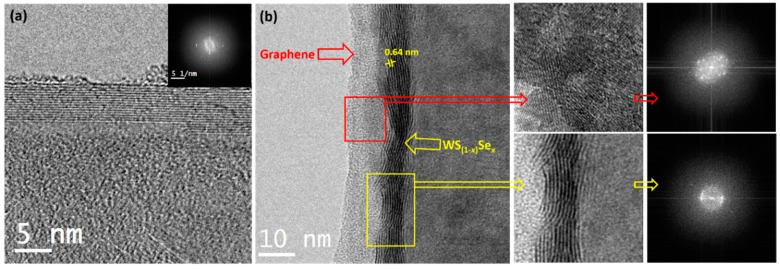
HRTEM images of (**a**) graphene/NF and (**b**) WS_(1−*x*)_Se*_x_*/graphene/NF. The layer spacing value is indicated as 0.64 nm, which is related to a (002) lattice plane d-spacing value. High-resolution HRTEM images show the corresponding graphene (red line) and WS_(1−*x*)_Se*_x_* (yellow line) lattice structures with selected area electron diffraction (SAED) pattern.

The surface morphological analysis was performed using a field emission scanning electron microscopy (FESEM) and a high-resolution transmission electron microscopy (HRTEM). Figure 2a–d shows the typical FESEM images of graphene, WS_2_/NF, WS_2_/graphene/NF, and WS_(1−*x*)_Se*_x_*/graphene/NF, respectively, and its insets show the higher magnification images. The bare NF (Figure 2a) is composed of lots of pores with sizes in tens to hundreds of micrometers, which will lead to somewhat irregular and uneven film growth which will be beneficial to enhance the HER property. From the FESEM image (Figure 2b), the regular deposition of graphene onto NF is observed with different thicknesses on the curvature of the foam. The larger area FESEM images of WS_2_/NF, WS_2_/graphene/NF, and WS_(1−*x*)_Se*_x_*/graphene/NF are provided in Figure S1. Wrinkles and ripples of graphene are spotted, which might be contributed to the differences in thermal expansion coefficients between the graphene and the Ni substrate [28]. The direct synthesis of WS_2_ on NF, for comparison, showed agglomerated small spherical granules with a non-uniform shape on the surface of the NF due to their curvature nature (Figure 2c). The nanoparticles with different sizes, due to agglomeration, were observed for WS_2_/graphene/NF from the low and higher magnification FESEM images (Figure 2d). In the case of WS_(1−*x*)_Se*_x_*/graphene/NF, the stacked nano-plate like agglomerated grains were observed as shown in Figure 2e. The elemental composition of WS_(1−*x*)_Se*_x_* alloys were determined by the energy dispersion spectra (EDS) as presented in Figure S2 (WS_(1−*x*)_Se*_x_* —W: 32.0%, S: 16.3%, C: 4.8%, and Se: 8.0%). Ni signals are ascribed from the NF substrate. The elemental mapping images of WS_2_ and WS_(1−*x*)_Se*_x_* layers are provided in Figures S3 and S4, and it confirms the homogeneous spatial distribution of W, S, C, and Se on the whole surface. The HRTEM studies were performed to reveal the layer structure of the prepared films. From the HRTEM images (Figure 3a), the multilayer of graphene was identified. In Figure 3b, WS_(1−*x*)_Se*_x_* and graphene are shown with yellow and red boxes, respectively.

Figure 2 and Figure 3 suggest the expose of active edge sites at the surface of WS_(1−*x*)_Se_*x*_/graphene/NF particles as reported in previous literature [23,29]. The WS_(1−*x*)_Se*_x_* nanosheets, with an interlayer separation of 0.64 nm, were grown intimately on the graphene/NF substrate, which are also beneficial to enhance a HER reaction.

**Figure 4:**
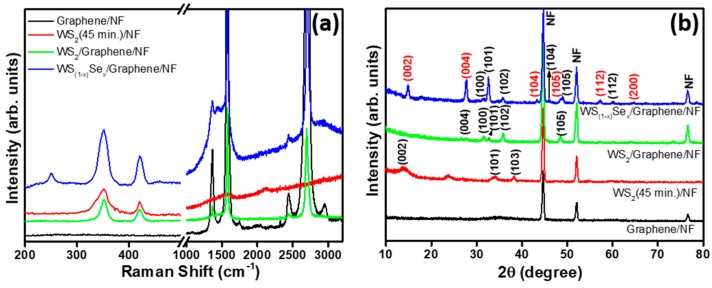
(**a**) Raman and (**b**) XRD spectra of graphene/NF, WS_2_(45 min)/NF, WS_2_/graphene/NF and WS_(1−*x*)_Se*_x_*/graphene/NF.

Raman spectroscopy was further used to characterize the formation of graphene, WS_2_/NF, WS_2_/graphene/NF, and WS_(1−*x*)_Se*_x_*/graphene/NF. From the spectrum of graphene/NF (Figure 4a), the principle bands of graphene, such as G band (1577cm^−1^), 2D band (2706 cm^−1^), and D band (1364 cm^−1^) were exhibited due to defects in the carbon lattice [30]. For WS_2_/NF, two prominent Raman peaks originated at 350.1 and 420.5 cm^−1^ correspond to the E^1^_2g_ and A_1g_ modes, respectively [31]. In the case of WS_2_/graphene/NF, E^1^_2g_, and A_1g_ modes (351.6 and 420.5 cm^−1^) for WS_2_, two sharp peaks for graphene (G band: 1577.8 cm^−1^ and 2D band: 2699.8 cm^−1^) are shown [32,33]. In addition to the above peaks, a low intensity E^1^_2g_ mode peak was observed at 250.6 cm^−1^, corresponding to WSe_2_ [34] for WS_(1−*x*)_Se*_x_*/graphene/NF. Our results also confirm the formation of both WS_2_ and WSe_2_ on porous graphene foam. Furthermore, the observed Raman results are well consistent with previously reported results of a WS_2_ and WSe_2_ materials system [34].

**Figure 5:**
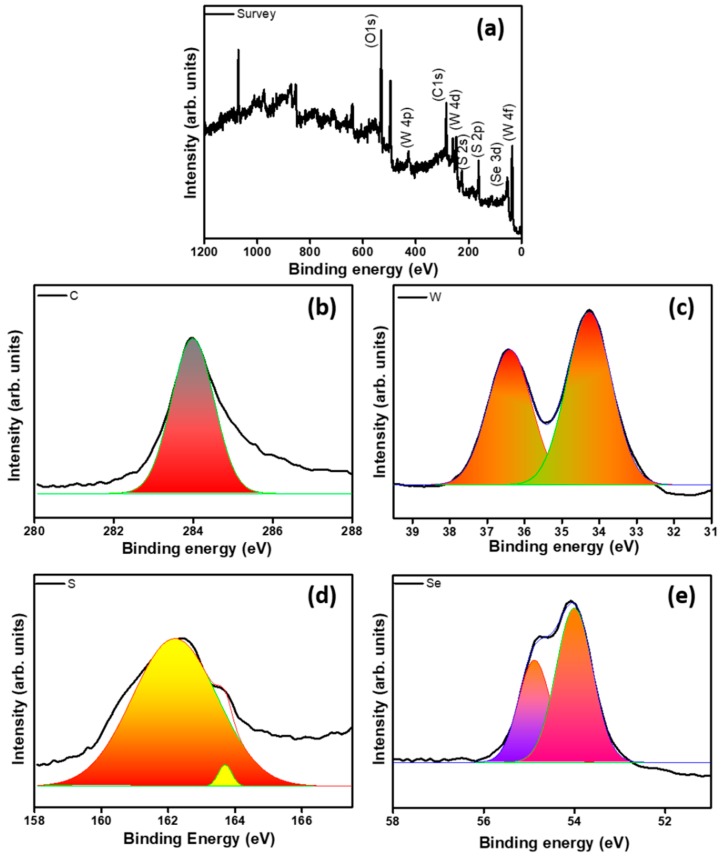
X-ray photoemission spectroscopy scan for WS_(1−*x*)_Se*_x_*/graphene/NF. (**a**) Survey scan; (**b**) C, (**c**) W; (**d**) S and (**e**) Se binding energies.

The structural and chemical composition of the graphene, WS_2_/NF, WS_2_/3D graphene, and WS_(1−*x*)_Se*_x_*/graphene/NF were further investigated via an X-ray diffraction (XRD) and X-ray photoelectron (XPS), respectively. The XRD patterns of graphene, WS_2_/NF, WS_2_/3D graphene, and WS_(1−*x*)_Se*_x_*/graphene/NF samples are shown in Figure 4b. No distinguishable diffraction signals were observed from graphene/NF due to their relatively low diffraction intensity. For WS_2_/NF (Figure 4b), the peaks appeared at 14.1°, 33.1°, and 38.3° that correspond to the (002), (101), and (103) lattice planes, respectively, which are consistent with hexagonally structured WS_2_ (WS_2_: JCPDS 657515). For WS_2_/graphene/NF, (004), (100), (101), (102), and (105) peaks were observed for WS_2_. In the case of WS_(1−*x*)_Se*_x_*/graphene/NF, WSe_2_ (002), (004), (104), (105), (112), and (200) lattice planes appeared in addition to the WS_2_/graphene/NF peaks as shown in Figure 3b (WSe_2_: JCPDS No. 89-5257). From the XPS survey scan of WS_2_/graphene/NF (Figure S5), the observation of C, Ni, W, and S elements was confirmed. For WS_(1−*x*)_Se*_x_*/graphene/NF (Figure 5a), an additional peak of Se element was detected. The expanded region of W4f, S2p, C, and Se3d peaks are provided in the Figure 5b–e. A sharp peak at 284 eV originated from graphene. The two principal peaks of W binding energy of 4f_7/2_ and W 4f_5/2_ (36.4 and 34.5 eV) doublets, which were indicative of the oxidation state of W^4+^, appeared. The S 2p_1/2_ and 2p_3/2_ orbital peaks observed at 163.6 and 161.9 eV, respectively, indicating the S2, confirmed the WS_2_ crystal [31,35]. The Se 3d core levels can be fitted with Se 3d_5/2_ (53.8 eV) and Se 3d_3/2_ (55.6 eV) corresponding to the −2 oxidation state of selenium [36].

**Figure 6:**
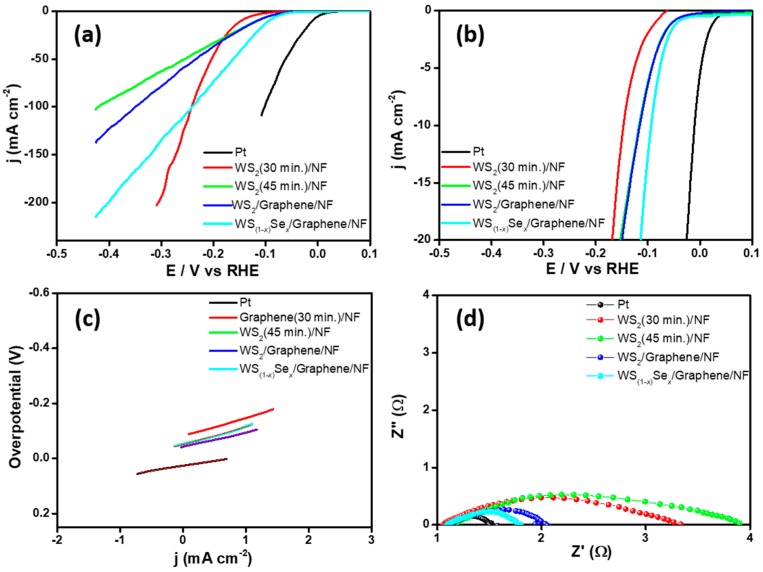
Electrochemical performance of different electrocatalysts. (**a**,**b**) Linear sweep voltammetry (LSV) curves of Pt, WS_2_(45 min)/NF, WS_2_/graphene/NF and WS_(1−*x*)_Se*_x_*/graphene/NF electrocatalyst with scan rate @ 10 mV s^−1^; (**c**) corresponding Tafel plots obtained from the LSV curves; (**d**) EIS spectra for Pt, WS_2_(45 min)/NF, WS_2_/graphene/NF and WS_(1−*x*)_Se*_x_*/graphene/NF electrocatalyst.

HER activities were investigated via a standard three-electrode setup with a scan rate of 10 mV s^−1^ in 0.5 M sulfuric acid (H_2_SO_4_) electrolyte solution by linear sweep voltammetry (LSV) with iR correction. As expected, the commercial Pt wire exhibited the lowest overpotential, which was close to zero. The WS_(1−*x*)_Se*_x_*/graphene/NF catalyst can deliver an overpotential at −93 mV vs. the reversible hydrogen electrode (RHE) for a geometric current density of 10 mA cm^−2^. In contrast, WS_2_(45 min)/NF, and WS_2_/graphene/NF exhibited inferior HER activity (−114 and −115 mV vs. RHE at current 10 mA cm^−2^, respectively) (Figure 6a,b). Earlier research reported that the unsaturated Se facets are highly active and improve HER activity [37,38]. Theoretical estimation supports lower Gibbs free energy for H_2_ adsorption onto the Se facets than the S facets [38]. Graphene is a conductive material which can increase the conduction between electrode and electrolyte and create the synergistic effect with active materials that lead to good HER properties.

The catalytic overpotential (−93 mV) of the WS_(1−*x*)_Se*_x_*/graphene/NF was quite lower than those of the reported WS_2_-based TMDCs in the literature, which include: Cobalt sulfide @WS_2_/carbon cloth (CC) hybrid catalyst (−97.2 mV@ 10 mA cm^−2^) [39], WS_2_/reduced graphene oxide hybrid nanosheets (−150 ~ −200 mV@10 mA cm^−2^) [40], MoS_2_-WS_2_ (−129 mV@10 mA cm^−2^) [41], WS_2_@hollow nitrogen-doped carbon nanofibers (−185 mV@10 mA cm^−2^) [42], graphdiyne-WS_2_ 2D-nanohybrid electrocatalysts (−140 mV@10 mA cm^−2^) [43], and Mo_(1−*x*)_W*_x_*S_2_ hollow nanospheres on an Ni_3_S_2_ nanorod (−98 mV@10 mA cm^−2^) [44]. For comparison, graphene on NF and WS_2_ (annealed at 30 min in an S environment) on NF were used as electrocatalysts, and their LSV curves are provided in the supporting information Figure S6 (overpotential of −193 and −145 mV vs. RHE, respectively).

The inherent property of catalytic activity for HER kinetics was probed by extracting the slopes from the linear regions in Tafel plots. A Tafel slope of 51 mV dec^−1^ was extracted for WS_(1−*x*)_Se*_x_*/graphene/NF, which is close to the value of a commercial Pt catalyst, and 62 mV and 63 mV dec^−1^ were obtained for WS_2_(45 min)/graphene/NF and WS_2_/NF, respectively (Figure 6c). The Tafel slope, 51 mV dec^−1^ of WS_(1−*x*)_Se*_x_*/graphene/NF, was lower than those of the previously reported WS_2_-based catalysts as well as other hybrid catalysts for HER, such as 3D WS_2_/graphene/Ni (87 mV dec^−1^) [45], nanostructured WS_2_/CC (127~105 mV dec^−1^) [46], bulk WS_2_ and WS_2_ nanosheets on bare oxidized carbon fiber (OCF) (149 ~ 99 mV dec^−1^) [47], WS_2_ @WS_2_ nanorattles, WS_2_ nanoflakes and bulk WS_2_ (68, 71 and 92 mV dec^−1^) [48], vertically-oriented WS_2_ nanosheet/graphene (73 mV dec^−1^) [49], WSe_2_ and WS_2(1−*x*)_Se_2*x*_ nanotubes (105 and 99 mV dec^−1^) [50], and monolayer of WS_2_ and WS_2(1−*x*)_Se_2*x*_ with a tunable band gap (100 and 85 mV dec^−1^) [51]. Previous research demonstrated the influence of Se inclusion to create the abundant active edges which can be promote HER properties and hence to perceive the low overpotential and small Tafel slope [52].

The exchange current density (*j*_0_) was found to be ~0.162, ~0.165, and ~0.274 mA cm^−2^, for WS_2_/NF, WS_2_/graphene/NF and WS_(1−*x*)_Se*_x_*/graphene/NF, respectively. The observed high *j*_0_ for WS_(1−x)_Se*_x_*/graphene/NF may be attributed to the large number of exposed active edge sites, good electrical conductivity, or the porous structure. The observed HER parameters for different electrodes are listed in Table 1. In acid solutions, three controlled reactions occur when hydrogen evolves on a metal chalcogenide catalyst. The overall HER reaction may proceed via a discharge step (Volmer-reaction, Equation (1)) followed by the ion-atom reaction (Heyrovsky reaction, Equation (2)) that leads to a Tafel slope of 40 mV dec^−1^, or a combination reaction (Tafel-reaction, Equation (3)) that leads to a Tafel slope of 30 mV dec^−1^.

(1) $$\;H_{3}O^{+}+e^{-}\to H_{\mathrm{ads}}+H_{2}O\;$$

(2) $$\;H_{\mathrm{ads}}+H_{3}O^{+}+e^{-}\to H_{2}+H_{2}O\;$$

(3) $$\;H_{\mathrm{ads}}+H_{\mathrm{ads}}\to H_{2}\;$$

From the classical theory for hydrogen evolution process, the observed Tafel slope of 36 mV dec^−1^ for Pt exposes the hydrogen production proceeds with the fast discharge step (Equation (1)) followed by the Tafel (Equation (3)) [53,54]. The observed intermediate Tafel slope values of 51, 62, and 63 mV·dec^−1^ (WS_2_/NF, WS_2_/graphene/NF and WS_(1−*x*)_Se*_x_*/graphene/NF, respectively) suggest that hydrogen production proceeds with the fast discharge step (equation 1) followed by the Tafel (Equation (3)) or Heyrovsky ion-atom reaction (Equation (2)) [17,55,56]. The observed small overpotential and Tafel slope for WS_(1−*x*)_Se*_x_*/graphene/NF could be attributed to the nanostructured particles on the porous substrate, which increase accessible active sites.

EIS was performed to study the interface reactions and electrode kinetics in HER at a frequency range from 0.01 Hz to 100 kHz. The Nyquist plots revealed the charge-transfer resistance (*R*_ct_) of Pt, WS_2_(45 min)/NF, WS_2_/graphene/NF, and WS_(1−*x*)_Se*_x_*/graphene/NF. The *R*_ct_ value of Pt, WS_2_(45 min)/NF, WS_2_/graphene/NF, and WS_(1−*x*)_Se*_x_*/graphene/NF were approximately 0.5, 2.4, 1.1, and 0.8 Ω, respectively (Figure 6d). The lower *R*_ct_ value suggests a faster reaction rate between the electrode and electrolyte. The low *R*_ct_ value could be due to the abundance of accessible sulfur/salinization active edges on a 3D porous substrate and result in the higher HER activity.

**Figure 7:**
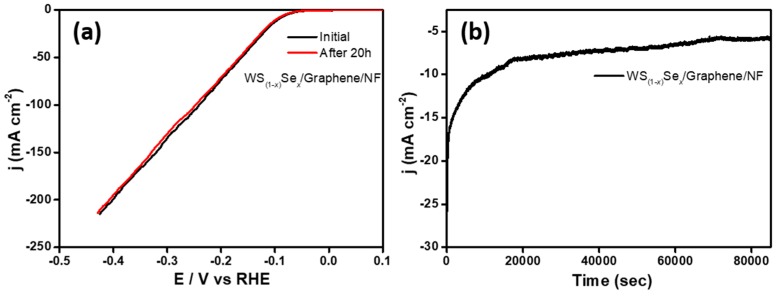
LSV curves for WS_(1−*x*)_Se*_x_*/graphene/NF before and after 20 h HER performance. Time dependent current density variation of WS_(1−*x*)_Se*_x_*/graphene/NF catalyst at a constant applied potential of −93 mV for more than 20 h.

Stability is another key factor to elucidate the performance of catalysts. For this purpose, we tested the stability of WS_(1−*x*)_Se*_x_*/graphene/NF electrode using potential cycling in the range from −0.5 to +0.1 V with a scan rate of 50 mV·s^−1^. After a 20 h operation in a 0.5 M H_2_SO_4_ solution, the polarization curve was little changed from the initial one, which indicated no observable degradation after long-term cycling tests (Figure 7a). The long-term electrochemical stability of this electrode was also examined. The cathodic current density for the WS_(1−*x*)_Se*_x_*/graphene/NF catalyst remained stable and exhibited no obvious degradation for electrolysis at a fixed overpotential of −93 mV for more than 20 h, which indicated the potential usage of this catalyst maintained its catalytic activity over a long time in the electrochemical process (Figure 7b).

## 4. Conclusions

In summary, an effective and efficient strategy was adopted for the synthesis of WS_2_ and ternary WS_(1−*x*)_Se*_x_*/graphene/NF for a robust and stable self-standing hydrogen evolving catalyst. The novel WS_(1−*x*)_Se*_x_*/graphene/NF catalyst showed good HER catalytic properties in acidic electrolyte with an overpotential of −93 mV to drive 10 mA cm^−2^, a small Tafel slope of 51 mV dec^−1^, and a high exchange current density with excellent long-term durability. Our results proved that Se incorporated WS_2_/graphene/NF exhibits the highest electrocatalytic activity for HER, and it is stable in acidic media over a long period among the other electrodes due to high active edge sites and porous structures.

## Figures and Tables

**Table 1:** Comparison of catalytic parameters of different HER catalysts.

Catalyst	Overpotential (mV vs. RHE) @10 mA cm^−2^	Tafel Slope (mV dec^−1^)	Exchange Current Density (*j*_0_, mA cm^−2^)
Pt	−10	36	5.98
WS_2_(45 min)/NF	−115	63	0.162
WS_2_/graphene/NF	−114	62	0.165
WS_(1−*x*)_Se*_x_*/graphene/NF	−93	51	0.274

## Supplementary Materials

The following are available online at http://www.mdpi.com/2079-4991/8/11/929/s1, Figure S1: Low magnification of FESEM images. (a) WS_2_(45 min)/NF; (b) WS2/graphene/NF and (c) WS_(1−*x*)_Se*_x_*/graphene/NF. Figure S2: EDS spectrum for WS_(1−*x*)_Se*_x_*/graphene/NF. Figure S3: (a) FESEM image of WS2/graphene/NF and its elemental mapping images of (b) Ni (c) W (d) S and (e) Se elements. Figure S4: (a) FESEM image of WS_(1−*x*)_Se*_x_*/graphene/NF and its elemental mapping images of (b) Ni (c) W (d) C (e) S and (f) Se elements. Figure S5: X-ray photoemission spectroscopy scan for WS_2_(45 min)/graphene/NF. (a) survey scan; (b) C; (c) W; and (d) S binding energies. Figure S6: Linear sweep voltammetry curves of graphene/NF and WS2(30 min.)/NF electrocatalyst.
