# Supplementary material for: WS(1−x)Sex Nanoparticles Decorated Three-Dimensional Graphene on Nickel Foam: A Robust and Highly Efficient Electrocatalyst for the Hydrogen Evolution Reaction
Source: Nanomaterials (Basel). 2018 Nov 8;8(11):929. doi: 10.3390/nano8110929 (PMC6266445; doi:10.3390/nano8110929)
Supplement: Supplementary file 1 [file nanomaterials-08-00929-s001.pdf]

Supporting Information

$WS_{(1-x)}Se_x$ Nanoparticles Decorated Three-Dimensional Graphene on Nickel Foam: A Robust and Highly Efficient Electrocatalyst for The Hydrogen Evolution Reaction

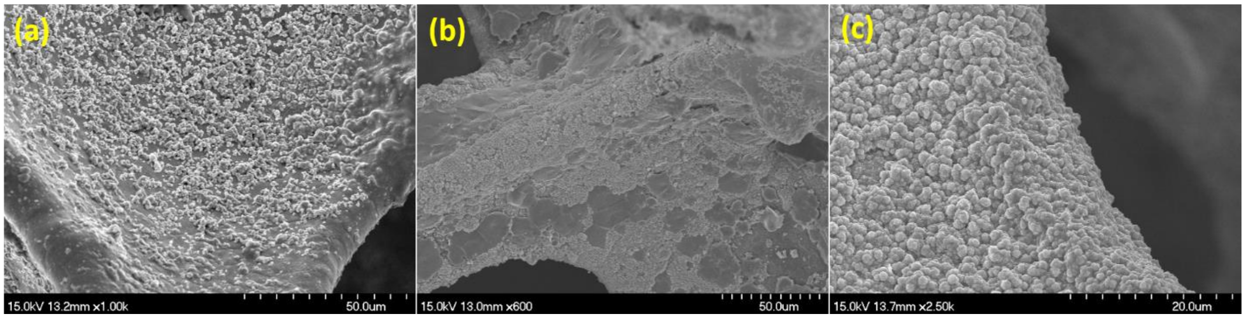

Figure S1. Low magnification of FESEM images. (**a**) WS_2(45 min.)/NF; (**b**)WS_2/graphene/NF and (**c**) $WS_{(1-x)}Se_x$/graphene/NF.

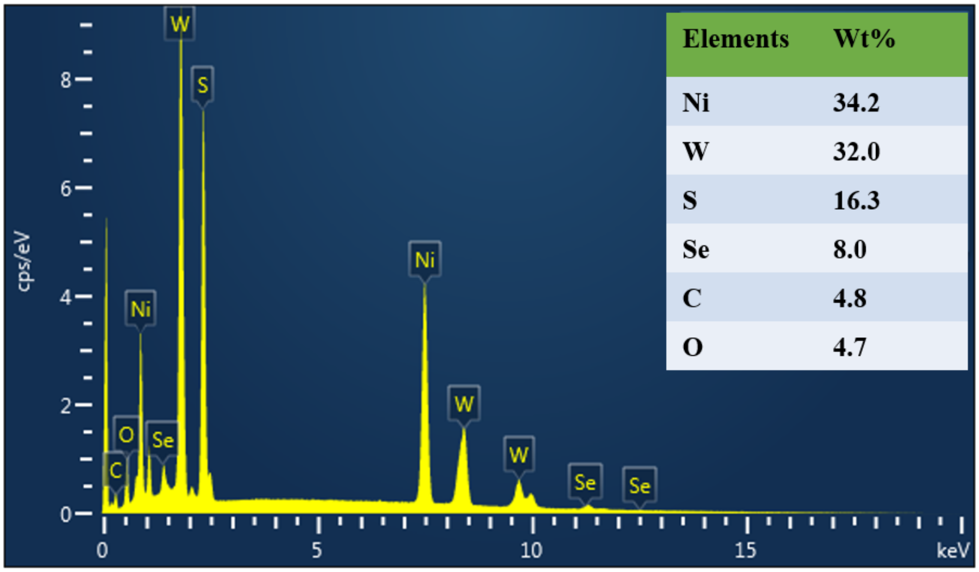

Figure S2. EDS spectrum for $WS_{(1-x)}Se_x$/graphene/NF.

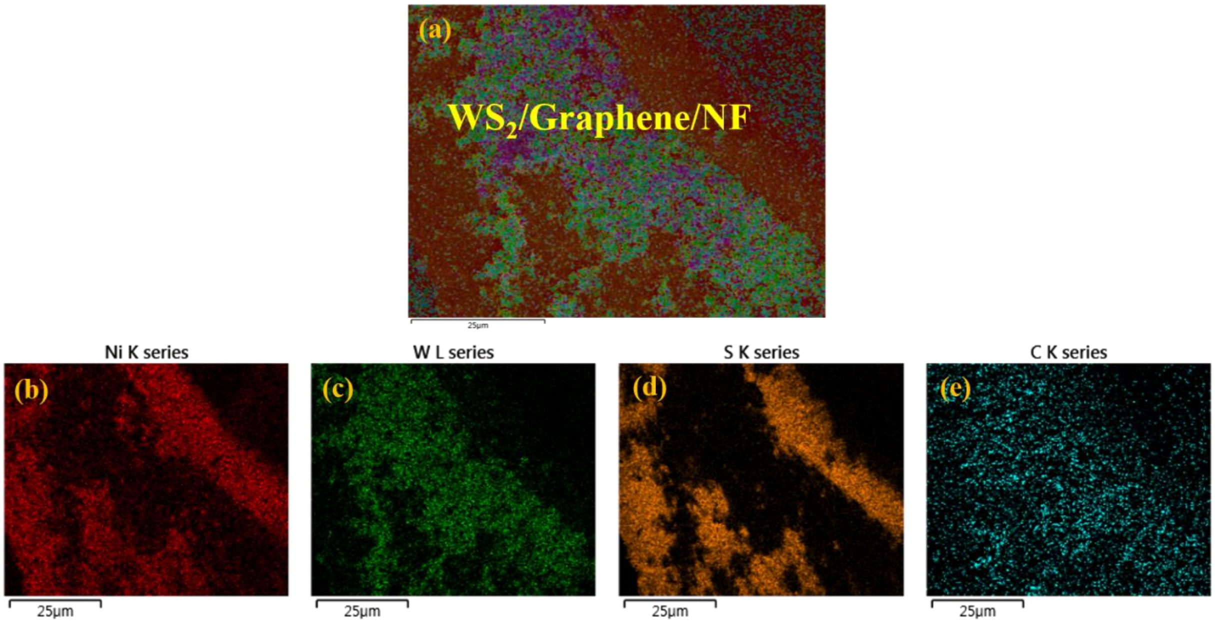

Figure S3. (**a**) FESEM image of WS_2/graphene/NF and its elemental mapping images of (**b**) Ni (**c**) W (**d**) S and (**e**) Se elements.

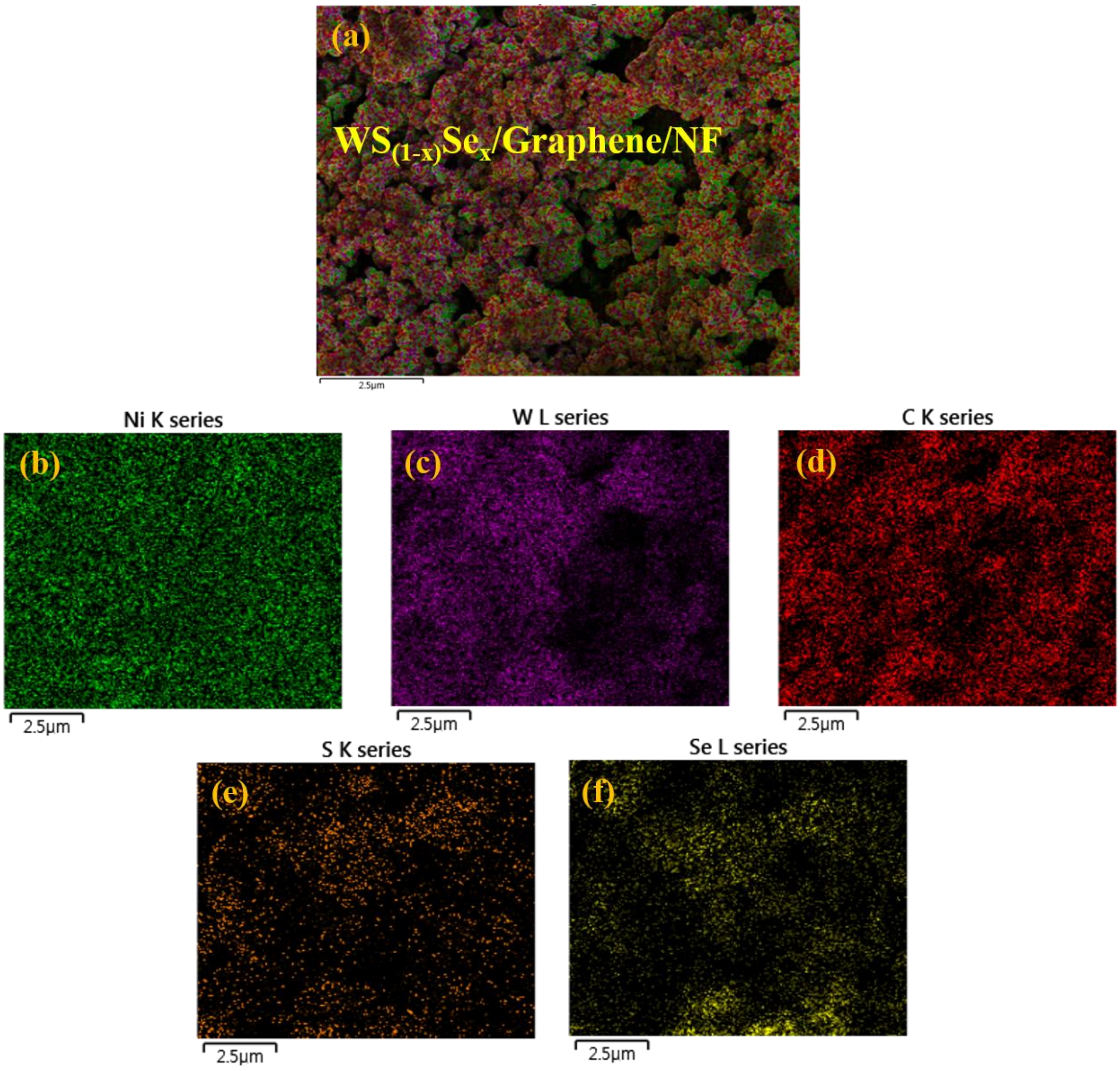

Figure S4. (**a**) FESEM image of $WS_{(1-x)}Se_x$/graphene/NF and its elemental mapping images of (**b**) Ni (**c**) W (**d**) C (**e**) S and (**f**) Se elements.

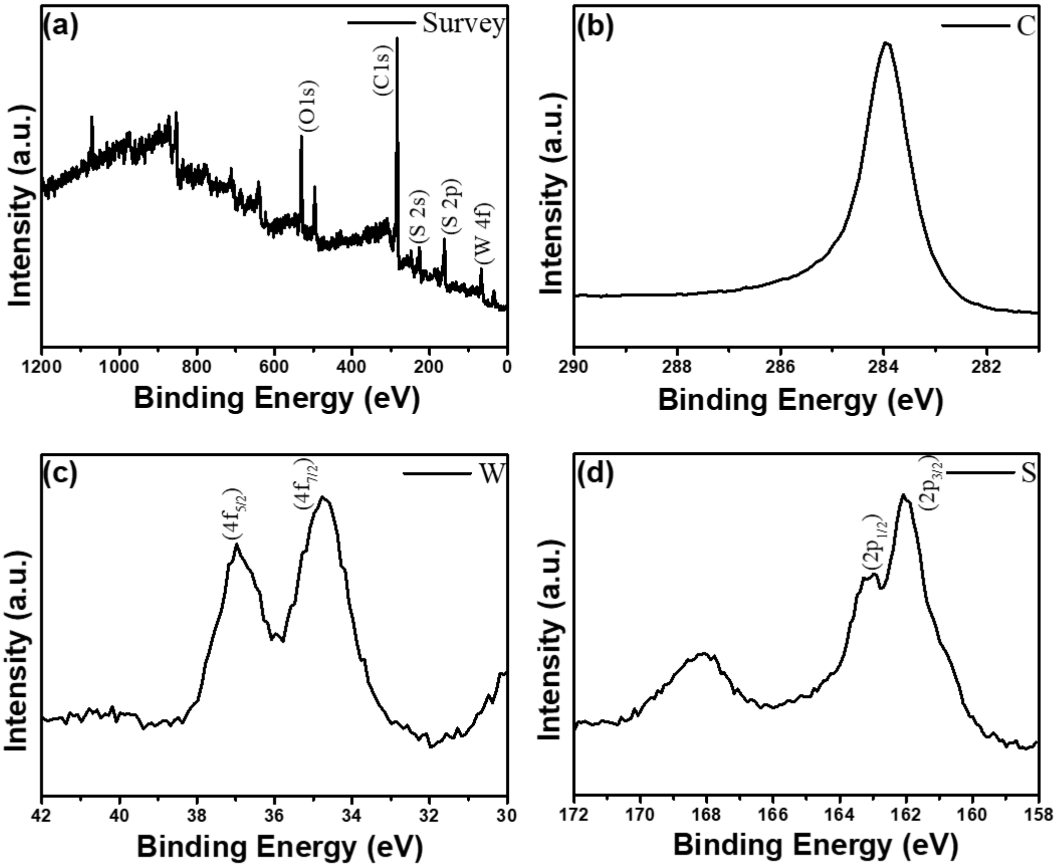

Figure S5. X-ray photoemission spectroscopy scan for WS_2(45 min)/graphene/NF. (**a**) survey scan; (**b**) C; (**c**) W; and (**d**) S binding energies.

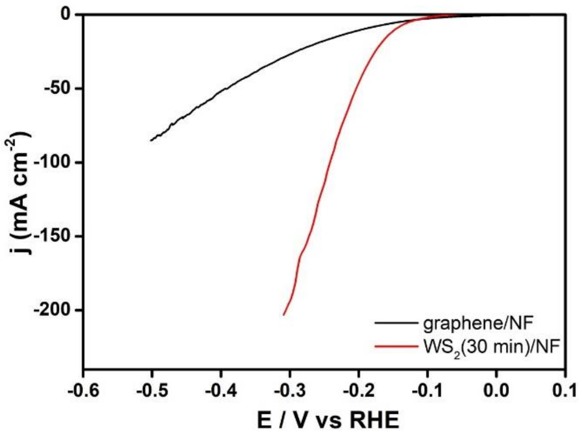

Figure S6. Linear sweep voltammetry curves of graphene/NF and WS_2(30 min.)/NF electrocatalyst.
